# Differential regulation of rho GTPases during lung adenocarcinoma migration and invasion reveals a novel role of the tumor suppressor StarD13 in invadopodia regulation

**DOI:** 10.1186/s12964-020-00635-5

**Published:** 2020-09-08

**Authors:** Maria Al Haddad, Rayane El-Rif, Samer Hanna, Leila Jaafar, Rayanne Dennaoui, Sandra Abdellatef, Veronika Miskolci, Dianne Cox, Louis Hodgson, Mirvat El-Sibai

**Affiliations:** 1 Department of Natural Sciences, School of Arts and Sciences, Lebanese American University, P.O. Box: 13-5053. Chouran, Beirut, 1102 2801 Lebanon; 2 Department of Pediatrics HemeOnc division, Weill Cornell Medicine, Joan & Sanford I. Weill Medical College of Cornell University, New York, USA; 3 Department of Medical Microbiology and Immunology, University of Wisconsin – Madison, Madison, WI 53706 USA; 4 Department of Anatomy and Structural Biology, Albert Einstein College of Medicine of Yeshiva University, Bronx, New York, USA; 5 Gruss-Lipper Biophotonics Center, Albert Einstein College of Medicine of Yeshiva University, Bronx, New York, USA

**Keywords:** StarD13, Rho GTPases, Invadopodia, Migration, Invasion

## Abstract

**Background:**

Lung cancer is the second most commonly occurring cancer. The ability to metastasize and spread to distant locations renders the tumor more aggressive. Members of the Rho subfamily of small GTP-binding proteins (GTPases) play a central role in the regulation of the actin cytoskeleton and in cancer cell migration and metastasis. In this study we investigated the role of the RhoA/Cdc42 GAP, StarD13, a previously described tumor suppressor, in malignancy, migration and invasion of the lung cancer cells A549.

**Methods:**

We knocked down StarD13 expression in A549 lung cancer cells and tested the effect on cell migration and invadopodia formation using time lapse imaging and invasion assays. We also performed rescue experiments to determine the signaling pathways downstream of StarD13 and transfected the cells with FRET biosensors for RhoGTPases to identify the proteins involved in invadopodia formation.

**Results:**

We observed a decrease in the level of expression of StarD13 in lung tumor tissues compared to normal lung tissues through immunohistochemistry. StarD13 also showed a lower expression in the lung adenocarcinoma cell line A549 compared to normal lung cells, WI38. In addition, the depletion of StarD13 increased cell proliferation and viability in WI38 and A549 cells, suggesting that StarD13 might potentially be a tumor suppressor in lung cancer. The depletion of StarD13, however, inhibited cell motility, conversely demonstrating a positive regulatory role in cell migration. This was potentially due to the constitutive activation of RhoA detected by pull down and FRET assays. Surprisingly, StarD13 suppressed cell invasion by inhibiting Cdc42-mediated invadopodia formation. Indeed, TKS4 staining and invadopodia assay revealed that StarD13 depletion increased Cdc42 activation as well as invadopodia formation and matrix degradation. Normal lung cells depleted of StarD13 also produced invadopodia, otherwise a unique hallmark of invasive cancer cells. Cdc42 knock down mimicked the effects of StarD13, while overexpression of a constitutively active Cdc42 mimicked the effects of its depletion. Finally, immunostaining and FRET analysis revealed the absence of StarD13 in invadopodia as compared to Cdc42, which was activated in invadopodia at the sites of matrix degradation.

**Conclusion:**

In conclusion, StarD13 plays distinct roles in lung cancer cell migration and invasion through its differential regulation of Rho GTPases.

Video abstract.

## Background

Lung cancer is the second most common cancer among both men and women. Its death rate amounts to around 26% of all cancer-related deaths, which exceeds the cumulative total of three of the deadliest cancer types: breast, pancreatic, and colon (Howlander, et al., 2017). Of the two types of lung cancer; small cell lung cancer (SCLC) and non-small cell lung cancer (NSCLC), NSCLC is the more common (Hayes, et al., 2006). In its most progressive stage, stage IV, NSCLC reaches both lungs infiltrates the area around them and metastasizes to distant organs (Chheang, et al., 2013; Tsim, et al., 2010).

Invasion and metastasis of lung cancer is a complex process that revolves around the acquired ability of the cancer cells to migrate [1, 2]. Cell migration is a well-choreographed series of events that integrates multiple intracellular signaling pathways. To actively migrate, the cell receives an initial chemoattractant signal, that defines it direction of migration, leading to the cell undergoing de novo actin polymerization in order to protrude towards the direction of the chemoattractant [3]. This is followed by the formation of adhesion structures that stabilize the protrusion [4], the locomotion of the cell body forward through contractile force, and finally the release of adhesion structures at the cell tail in order to propel the cell in the direction of motility [5]. These processes are largely regulated by the Rho family of small guanosine triphosphatases (GTPases), which includes key enzymes that play a major role in the reorganization of the actin cytoskeleton [6]. Indeed almost all processes and events of tumor cell proliferation, motility and invasion including cellular polarity, cytoskeletal re-organization, and signal transduction pathways are controlled through the interplay between the different Rho-GTPases [7–10].

Rho GTPases are small monomeric G proteins that belong to the Ras superfamily [11]. The three most characterized members are RhoA, Rac1, and Cdc42 [12]. Rho GTPases are found in two forms, a GDP-bound inactive and a GTP-bound active form [13]. Rho GTPases are regulated by three classes of proteins, Guanine nucleotide exchange factors (GEFs), GTPase-activating proteins (GAPs), and guanine nucleotide dissociation inhibitors (GDIs). GAPs negatively regulate Rho GTPases by stimulating the intrinsic GTPase activity of Rho GTPases and promoting the formation of the inactive GDP-bound form [14].

StarD13 (Steroidogenic acute regulatory protein-related lipid transfer domain-containing protein 13 (StarD13), also known as *DLC2*, is a GAP for RhoA and Cdc42 [15]. The *DLC2* gene, located on position *13q12.3* was first identified by Ching et al. (2003). StarD13 has an N-terminal SAM motif and a C-terminal START domain. It also harbors a RhoGAP domain, which is important to its function [16–18]. Researchers have shown that StarD13 is underexpressed in hepatocellular carcinoma and that the overexpression of StarD13 significantly decreases cell growth and proliferation [18]. Moreover, DLC1, a closely related protein, is underexpressed in many types of cancer including lung, prostate, kidney, colon, breast, uterus, and stomach [19]. Furthermore, a Genome-Wide Analysis integrating a paired copy number and gene expression survey on glioblastoma samples concluded that StarD13 is a potential tumor suppressor gene that could be involved in the resistance of this tumor type to etoposide [20]. Also, previous data in astrocytoma, breast cancer and colon cancer, suggested the role of StarD13 as a tumor suppressor in these cancers, while revealing a simultaneous positive regulatory role in cell migration [21–24]. Indeed, when StarD13 was silenced, the associated constitutive activation of RhoA (and indirect inhibition of Rac1) inhibited cell migration. This was the result of the excessive attachment of cells to the substratum which disrupts their ability to move forward [22, 23].

This study characterizes the role of StarD13 in lung adenocarcinoma cells survival. It also examines the pathways downstream from StarD13 that regulate lung cancer cell migration and invasion. Data obtained from analyzing the expression levels of StarD13 in patient tissues compared to normal tissues revealed an underexpression of StarD13 in lung cancer, as has been described for other tumor types. In addition, here we demonstrate a key inhibitory role for StarD13 in the regulation of cellular proliferation in normal and cancer lung cells. Conversely, StarD13 was required for lung cancer cell migration. Mechanistically, StarD13 effects on cancer cell motility and adhesion in vitro were mediated by its RhoA GAP activity on RhoA and its indirect Rac1 regulation*.* Finally, and for the first time, we demonstrate a role for StarD13 in invadopodia formation, matrix degradation and invasion of lung adenocarcinoma cells by regulating Cdc42.

## Methods

### Cell culture

Human Non-small cell lung adenocarcinoma cell line A549 and human normal lung cells WI38 (obtained from ATCC) was cultured in DMEM medium supplemented with 10% FBS and 100 U penicillin/streptomycin at 37 °C and 5% CO_2_ in a humidified chamber.

### Antibodies and reagents

Rabbit polyclonal anti-StarD13 (used for western blots), mouse monoclonal anti-RhoA, mouse monoclonal anti-Rac1, rabbit polyclonal anti-Vinculin, rabbit polyclonal anti-Tks4, and rabbit polyclonal anti-beta actin were purchased from abcam (Abcam Inc., Cambridge, UK). Goat polyclonal anti-StarD13 (used for immunohistochemistry) mouse monoclonal anti-Cdc42 (used for Immunofluorescence) and rabbit polyclonal anti-Cdc42 (used for western blots) antibodies were purchased from Santa Cruz (Santa Cruz Inc., Delaware, CA, USA). Anti-rabbit and anti-mouse HRP-conjugated secondary antibodies were obtained from promega (Promega Co., Wisconsin, USA). Alexa488 and Alexa569-conjugated Goat anti-mouse and anti-rabbit IgG (H + L) were obtained from Invitrogen (Invitrogen, Waltham, MA, USA). Alexa 405-conjugated Goat anti-rabbit was obtained from abcam (Abcam Inc., Cambridge, UK). The GFP-Cdc42-Q61L, GFP-pcDNA3,1, GST-RBD (Rhotekin binding domain) and GST-CRIB (Cdc42 and Rac interactive binding domain) were a generous gift from Dr. Jonathan Backer (Albert Einstein College of Medicine, New York, USA), the Rac-Q61L and Cdc42-Q61L (constitutively active) constructs were a generous gift from Dr. Hideki Yamaguchi (Sasaki Foundation, Tokyo, Japan), the FRET-RhoA biosensor was a generous gift from Dr. Klaus Hahn (University of North Carolina Chapel Hill, NC, USA) [25, 26]. The Cherry-RhoA-CA (constitutively active) construct and the FRET-Cdc42 biosensor [27] were a generous gift from Dr. Louis Hodgson (Albert Einstein College of Medicine, New York, USA).

### Cell transfection and small interfering RNA

Human FlexiTube siRNA for each of [StarD13, oligos 3 and 8 out of the NM_001243466 panel] (target sequence: 5′-CCCGCAATACGCTCAGTTATA-3′), Rac1 [oligo 5 out of the NM_006908, NM_018890 and NM_198829 panels] (target sequence: 5′-ATGCATTTCCTG GAGAATATA-3′.), Cdc42 [oligo 4 out of the NM_001039802, NM_001791 and NM_044472 panels] and RhoA [oligo 6 out of the NM_001664 panel] (target sequence: 5′-TTCGGAA TGATGAGCACACAA-3′) were brought from Qiagen (Qiagen, USA). The cells were transfected with the siRNA at a final concentration of 10 nM using HiPerfect (Qiagen, USA) as per the manufacturer’s recommendations. Control cells were transfected with siRNA sequences targeting GL2 Luciferase (Qiagen, USA). After 72 h, protein levels in total cell lysates were analyzed by western blotting using the appropriate antibodies or the effect of the corresponding knock down was assayed. Western blot analyses were performed to choose the oligos showing the most effective silencing from every FlexiTube panel corresponding to the target sequence of proteins mentioned above in these cells. These oligos were used in this study and western blots showing efficiency of knock down are included in the study.

Cells transfected with both siRNA and a vector were incubated with the siRNA for 72 h as previously described, then co-transfected with the appropriate vector 24 h prior to the experiment. Cells were transfected with 5 μg of Rho-DA, Rac-DA, Cdc42-DA or control empty vector (pcDNA3.1) using Lipofectamine LTX with Plus reagent (Waltham, MA, USA) as described by the manufacturer.

### Pull down assays and Western blots

Cell lysates were collected from lung cancer cells treated with the various conditions for the indicated time points. The RhoA/Rac1/Cdc42 Activation Assay Combo Kit (Cell BioLabs, Sand Diego, CA, USA) was used for pull down assay following the manufacturer’s instructions. Briefly, cell lysates were incubated with GST-RBD (for Rho pull down) or GST-CRIB (for the Rac1 and Cdc42 pull down) for 1 h at 4 °C with gentle shaking. Then, the samples were centrifuged, and the pellet washed for several times. GTP-RhoA or GTP-Rac1/Cdc42 were detected by western blotting using anti-RhoA, anti-Rac1 or anti-Cdc42, respectively. Total RhoA/Rac1 and Cdc42 were collected prior to the incubation with GST-RBD/GST-CRIB and used as a loading control. For the Western blot, protein samples were separated by SDS-PAGE, transferred to PVDF membranes and the proteins of interest detected after incubating the membrane with the corresponding primary antibodies. Secondary antibodies were visualized with chemiluminescent reagent ECL (GE Healthcare). The results were obtained using the Chemidoc imaging system. The levels of protein expression were compared by densitometry using ImageJ (National Institutes of Health, MA, USA). In western blot experiments, the data were presented normalized to the actin loading control. In the Pull-down assays, the data were presented as fold change to the control condition (compared to experimental).

### Cell viability

A549 cells were seeded in 96-well plates (growth area: 0.6cm^2^) at a concentration of 1x10^6^cells/ml. Following treatment, 10 μl of Cell Viability Reagent (WST-1; Roche, Germany) was added to each well. Colorimetric quantitation of formazan was quantified at 450 nm. The absorbance of the each blank well was subtracted from the corresponding sample well. The results were normalized to the control and the fold change in proliferation was reported.

### Cell cycle analysis and Annexin staining

Treated cells were centrifuged at 1500 rpm for 5 min. The pellet was washed with 1 ml of ice-cold 1X phosphate-buffered saline (PBS) and resuspended in 4 ml of 70% ethanol. Cells were stained with 10 μl of propidium iodide (PI) for 10 min in the dark and cell cycle distribution analysis was performed using an Accuri C6 flow cytometer (Ann Arbor, MI, USA). The distribution of cells into their respective cell cycle phases based on their DNA content was determined by the CFlow® software. G0/G1 cells were 2n, S-phase cells were > 2n but <4n while G2/M cells were 4n.

For Annexin staining, treated cells were stained with 5 μl of Annexin V-FITC and 10 μl of propidium iodide cells, and incubated in the dark at room temperature for 10 min. The fluorescence was determined immediately with the flow cytometer.

### Sphere formation assay

A549 cells were trypsinized and chilled on ice before mixing 2000 cells/condition with thawed Matrigel (Corning) in a 1:1 ratio. Following, 100 μl of the master mix (Cells + Matrigel) were pipeted in a circular manner around the rim of the wells ofa 24 well-plate and incubated at 37 °C for 45–60 min for the Matrigel to solidify. Cells were then transfected with siRNA. After 3 days, the media was replenished and the transfection master mix added again. This process was repeated every 72 h for 13 days. The diameter and area of spheres imaged by inverted light microscopy (10X objective) were quantified using the ZEN software. At least 50 spheres per condition were quantified, and the averaged values were used to draw the corresponding graphs. The volume and area of the spheres was calculated in excel.

### Antigen retrieval and immunohistochemistry

Paraffin-embedded normal and tumor lung tissue microarray was purchased from Capital Bioscience. Two rounds of deparaffinization of the tissues were performed by soaking the slides in Xylol for 20 min and 5 min, respectively. Following, the samples were hydrated by consecutive washes using ethanol 100% (twice for 5 min), ethanol 90% and ethanol 80%. The paraffin was removed by rinsing with tap water for 10 min, after which the tissues were re-hydrated by soaking in PBS for 20 min at RT. Meanwhile 200–300 ml of antigen retrieval buffer (1 mM EDTA pH = 8) was heated in a 700 W microwave for 5 min and added to the slides which were also microwaved twice for 5 min with a 1 min break and addition of cool retrieval buffer in between. After cooling down, the tissues microarray were surrounded with a water repellent material (DAKO pen, clear nail polish) and blocked for 30 min at RT in a 4% Blocking solution (PBS + 4% BSA + 0.1% Triton-X 100). The slides were incubated with anti-StarD13 antibody (and DAPI). Then with a secondary antibody coupled to Alexafluor-488 fluorophore. Tissue fluorescent images were taken on a Zeiss LSM confocal microscope (10x objective) (Zeiss, Oberkochen, Germany). All digital images were imported to ImageJ software for analysis (National Institutes of Health, MA, USA). We analyzed the total fluorescence intensity of a fixed area from at least 10 different frames from each tissue sample.

### Immunostaining and invadopodia assay

For immunostaining experiments, A549 lung cancer cells were plated on glass coverslips and transfected with the constructs or siRNA oligos for 72 h. For invadopodia assays, the cells were transfected then plated on Alexa 568-conjugated matrix-coated glass coverslips [28] (QCM Gelatin Invadopodia Assay-Millipore, MA, USA) for 48 h to measure the total degradation. For individual invadopodia detection, the cells were plated on Alexa 568-conjugated matrix for 8 h only. Next, the cells were fixed with 4% paraformaldehyde for 10 min at 37^ο^C and permeabilized with 0.5% Triton-X 100 for 15 min on ice. Following, the samples cells were blocked in 1% BSA solution for 1 h, before incubation with primary antibodies overnight at 4^ο^C, and with fluorophore-conjugated secondary antibodies for 1 h. Fluorescent images were taken using a 63X objective lens on Zeiss Observer Z1 microscope operated by the Zen software (Zeiss, Oberkochen, Germany).

To quantitate focal adhesions and invadopodia, two main plugins in Image J (National Institutes of Health, MA, USA), CLAHE and Log3D were used. CLAHE and Log3D. This enhances the contrast and filters the image based on user predefined parameters which allows the detection and analysis of focal adhesions [29]. Areas of focal adhesions (seen by vinculin staining) in different samples were presented as fold difference relative to the control. The number of invadopodia (seen by TKS4 staining) was expressed as absolute values of the means in every sample (from 3 experiments).

To quantitate matrix degradation in the invadopodia assay, we used image J. Traces of cells performed in DIC were transferred to the Alexa channel image (fluorescent matrix), and the mean fluorescent intensity/cell surface area was measured. A similar region of interest (ROI) was traced in the background of each cell and the mean intensity of the background was measured. Matrix degradation index obtained by dividing the fluorescent intensity in the background ROI to that in the cell trace was expressed as fold difference to reflect the degradation activity by the cell (from 3 experiments).

### FRET biosensor imaging and analysis

A549 cells plated on MatTek dishes were transfected with 2.5 μg of the RhoA fluorescence resonance energy transfer (FRET)-based biosensor [25] or the Cdc42-based FRET biosensor [27] using Lipofectamine LTX with Plus reagent (Invitrogen, US) as described by the manufacturer. Both biosensors show an increase in FRET ratio with GTP loading of RhoA or Cdc42, respectively. FRET images were obtained using a 63X objective lens on Zeiss Observer Z1 microscope supplemented with a computer-driven Roper cooled CCD camera and operated by Zen software (Zeiss, Oberkochen, Germany). A CFP/YFP FRET filter cube was used: YFP was imaged with exciter S500/20 and emitter S535/30 (YFP/acceptor image), CFP was imaged with exciter S430/25 and emitter S470/30 (CFP/donor image) or S535/30 (FRET image). Images backgrounds were corrected and the YFP images were thresholded to generate a binary mask with values of 1 for inside the cell and 0 for the background. Moreoever, we multiplied CFP and FRET images by the mask to remove the background from ratio calculations.. The increase in FRET signal due to activation of RhoA or Cdc42 was determined by dividing the FRET image (CFP excitation- YFP emission) by the donor image (CFP excitation- CFP emission). FRET signals were quantified by averaging the mean FRET ratio in all the cell area, normalizing the values to control cells (untreated) and expressing the difference as fold change. Detailed description of the image analysis process can be found in our previous publications [30].

### Wound healing assay

A549 cells were grown to confluence and treated as indicated. After 24 h, a wound was made in the monolayer with a sterile pipette tip, the cells were washed to remove debris and new low-serum medium (containing 0.5% FBS) was added. Phase-contrast images of the wounded area were captured at 0 and 72 h after wounding using the 10X objective of a Zeiss Observer Z1 microscope operated by the Zen software (Zeiss, Oberkochen, Germany).. Wound widths were measured at 11 different points for each wound, and the average rate of wound closure was calculated (in μm/hr) using the ImageJ software.

### Random motility assay

For motility analysis, images of cells moving randomly in serum were collected every 60 s for 2 h using a 20X objective of a Zeiss Observer Z1 microscope supplemented with a computer-driven Roper cooled CCD camera and operated by Zen software (Zeiss, Oberkochen, Germany). During imaging, the temperature was controlled using a Nikon heating stage set at 37 °C. The medium was overlayed with mineral oil and buffered using HEPES. The total distance travelled by individual cells, as well as their net paths, were measured in the ImageJ software using the ROI tracker plugin written by Dr. David Entenberg. The speed (μm/min) of at least 15 cells per condition was calculated by dividing the distance over time (120 min). The net distance travelled by the cell was also calculated by measuring the distance travelled between the first and the last frames. .

### Invasion assay

Invasion assay was performed 48 h following treatment using the collagen-based invasion assay (Millipore, Burlington, MA, USA) according to manufacturer’s instructions. Briefly, cells were starved with serum-free medium for 24 h. Following, the cells were harvested, and resuspended in serum free quenching medium to a final concentration of 10^6^cells/ml. Inserts were then placed in a 24-well plate, and 500 μl of complete media (with 10% serum) was added to the lower wells. The inserts were then stained for 20 min at room temperature with 400 μl of cell stain provided with the kit. Unbound stain was washed off and the remaining stain was extracted with extraction buffer. Finally, 100 μl of extracted stain was transferred to a 96-well plate suitable for colorimetric measurement using a plate reader. The optical density was measured at 560 nm. The data is presented as fold difference compared to control.

### mRNA expression analysis

To determine the expression of StarD13 in human lung tumors, we mined the publicly available Repository Oncoming gene expression microarray database (National Cancer Institute, https://www.oncomine.org/resource/login.html). Data was plotted using the normal versus lung cancer data sets and parameters and the threshold was set at *p*-value of 0.001.

### Statistical analysis

All the results reported represent average values from three independent experiments. All error estimates are given as ± SEM. The *p*-values were calculated by unpaired independent t-tests or chi-square tests depending on the experiment using the VassarStats: Website for Statistical Computation (http://vassarstats.net/). All results showed statistical significance with a *p*-value 0.05.

## Results

### StarD13 is a tumor suppressor in lung adenocarcinoma

**Fig. 1:**
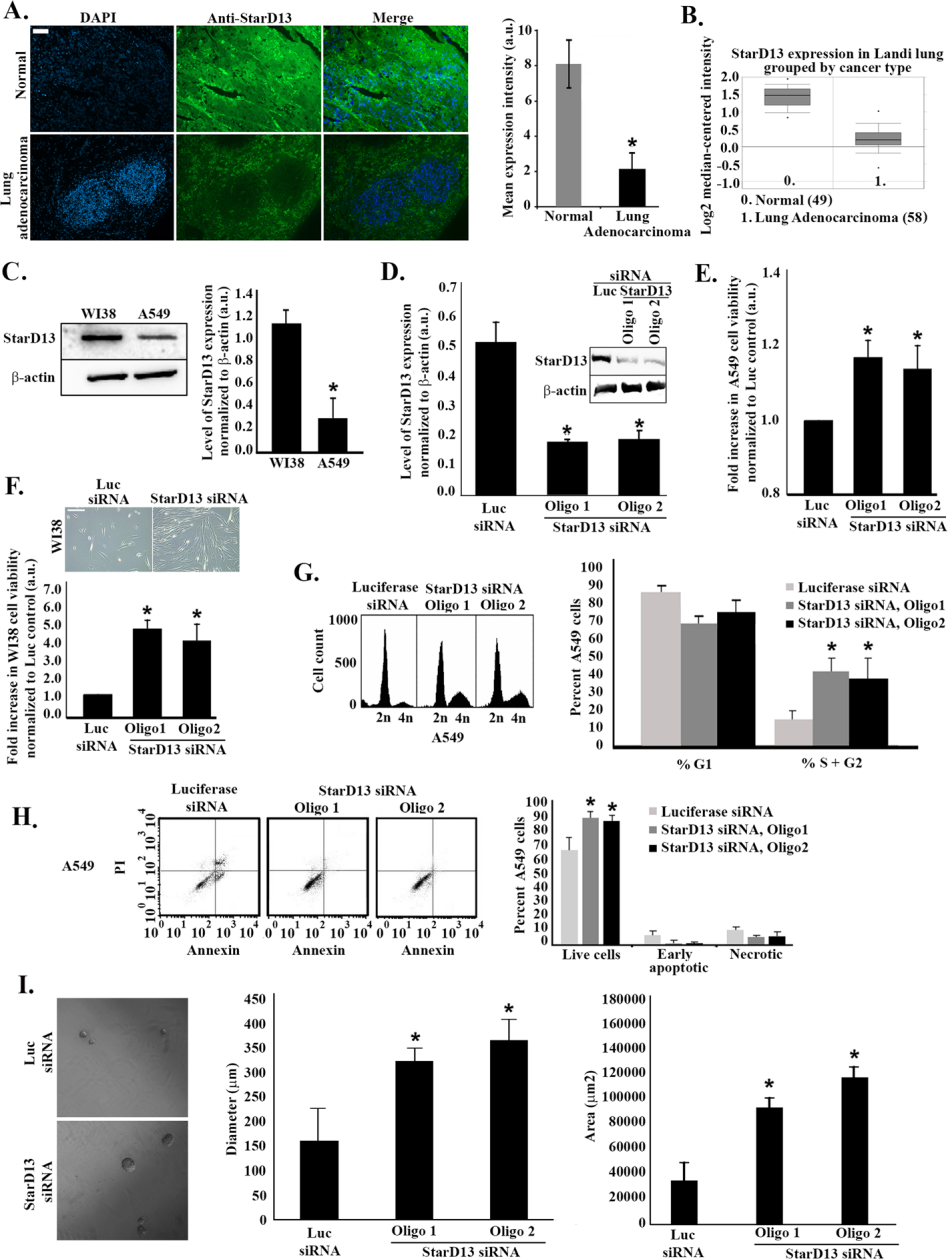
StarD13 is a potential tumor suppressor in lung adenocarcinoma. **a** Representative fluorescent micrographs of formalin-fixed normal lung tissue from biopsy (upper) or lung adenocarcinoma tissue (lower), paraffin embedded, sectioned and then stained with DAPI (left panels) and immunostained with anti-StarD13 antibody (middle panels). The right hand panels show the merged channels. The graph is a quantitation of the immunohistochemistry. The mean fluorescent intensity/pixel was measured using ImageJ software and expressed to the corresponding tissues. Data are the mean −/+ SEM from 3 different experiments (with 4 tissues each) and *p < 0.05. Scale bar is 100 μm. **b** Data analyzed from Oncomine website. mRNA of the indicated number of samples (indicated in the legend) were quantified for expression levels of StarD13 in Landi Lung. **c** A549 and WI38 cells were lysed and immunoblotted by western blot analysis for StarD13 (upper gel) or for β-actin (lower gel) for loading control. The graph is a quantitation (using the ImageJ software) of the bands from the StarD13 gel normalized to the corresponding bands in the actin gel. Data are the mean −/+ SEM from 3 different experiments and *p < 0.05. **d** A549 cells were transfected with luciferase control siRNA or with 2 oligos of StarD13 siRNA for 72 h. The **c**ells were then lysed and immunoblotted by western blot analysis for StarD13 (upper gel) or for β-actin (lower gel) for loading control. The graph is a quantitation (using the ImageJ software) of the bands from the StarD13 gel normalized to the corresponding bands in the actin gel. Data are the mean −/+ SEM from 3 different experiments and *p < 0.05. **e** Cell proliferation was determined using WST-1 reagent. Cell viability of siRNA-transfected A549 cells was expressed as fold increase from control (luciferase-transfected). Data are the mean −/+ SEM from 3 different experiments and *p < 0.05. **d** The micrographs are phase contrast representative images of luciferase siRNA- or StarD13 siRNA-transfected WI38 cells grown in culture for 72 h. Cell proliferation for siRNA-transfected WI38 cells was determined using WST-1 reagent and cell viability expressed as fold increase from control (luciferase-transfected). Data are the mean −/+ SEM from 3 different experiments and *p < 0.05. **g** A549 cells were transfected with luciferase or StarD13 siRNA permeabilized and stained with 30 μL propidium iodide for 10 min. Cells were analyzed using a C6 flow cytometer, which indicated the distribution of the cells into their respective cell cycle phases based on their DNA content. G0/G1 cells were 2n; S-phase cells were > 2n but <4n while G2/M were 4n. Cell DNA content was determined by CellQuest software. The graph is a quantitation expressed as percentage of cells in G1 and combined percentage of cells in S + G2. Data is the mean +/− SEM from 3 different experiments. **h** A549 cells were transfected with either control luciferase siRNA or with StarD13 siRNA. The cells were then trypsinized and stained with 5 μL of Annexin V FITC and 10 μL of Propidium Iodide. The fluorescence of the cells was determined immediately with a flow cytometer. Cells, which are early in the apoptotic process, will stain with the Annexin V FITC Conjugate alone. Live cells will show no staining by either the Propidium Iodide solution or Annexin V FITC conjugate. Necrotic cells will be stained by both the Propidium Iodide solution and Annexin V FITC conjugate. The graph is a quantitation expressed as percentage of cells. Data is the mean +/− SEM from 3 different experiments and *p < 0.05. **i** A549 transfected with Luciferase or StarD13 siRNA were grown in suspension and mixed with Matrigel to form spheres, as described in the Materials and Methods. The graphs are a quantitation of sphere diameter (μm) and area (μm^2^) of the spheres. Data is the mean +/− SEM from 50 spheres/condition and *p < 0.05

To investigate the tumor suppressor function of StarD13 in lung cancer, we examined its expression levels in lung cancer and normal lung biopsy tissue sections obtained from patients with Stage II Non-small cell lung adenocarcinoma. Immunohistochemistry using an anti-StarD13 antibody showed a significant decrease (4 fold) in StarD13 expression in lung tumor tissues as compared to normal biopsies (Fig. 1a).

The results in Fig. 1a were also confirmed by mining the Oncomine database for microarray analysis of StarD13 expression levels in different tumor types from four datasets (Landi lung, Garber lung, Hou lung and Su lung). The data consistently showed that StarD13 mRNA levels are lower in tumors relative to non-tumor tissues (Fig. 1b and Supplemental Figure S1), as is typically the case for tumor suppressors.

Consistently, western blot analysis revealed a significant underexpression (higher than 4 fold) of StarD13 in A549 lung cancer cells compared to the normal lung cells WI38 (Fig. 1c).

Next, we depleted StarD13 using small interfering siRNA and confirmed the decrease in the expression levels by western blot. Figure 1d shows that all four oligos tested, were successful in targeting StarD13 with oligo 3 and oligo 8 exhibiting the highest StarD13 knock down efficiency (around 60%) as compared to control cells transfected with luciferase. These oligos were then used for all subsequent experiments in the study and referred to as oligos 1 and 2.

To determine the anticancer potential of StarD13, we used the WST-1 assay and compared the viability of control and StarD13 depleted A549 cancer cells (Fig. 1e). As expected, cell viability significantly increased (15 to 20%) in cells depleted of StarD13 as compared to the control. The same anti-cancer effect was observed in WI38 normal lung cells, where cell viability significantly increased (around 5 fold) in WI38 cells depleted of StarD13 as compared to the control, further suggesting the tumor suppressor role of StarD13 in lung (Fig. 1f).

The effect of StarD13 on cell viability and cell proliferation was further investigated by examining the impact of StarD13 silencing on cell cycle distribution and cell death by flow cytometry. Staining of A549 cells with propidium iodide alone showed that, upon StarD13 depletion, cells undergo more cell division, as evidenced by the 30% increase in the percentage of A549 cells in the S and the G2/M phases as compared to the control (Fig. 1g). Staining of A549 cells with both AnnexinV-FITC and PI showed that StarD13 knock down increases the percentage of viable cells (negative for both AnnexinV-FITC and PI) by approximately 20% (Fig. 1h). Finally, a sphere formation assay revealed the formation of larger spheres of A549 cells in matrigel (higher sphere area and diameter), secondary to the StarD13 depletion, which is indicative of increased cell division (Fig. 1i). Collectively, the results presented in Fig. 1 demonstrate that StarD13 acts as a tumor suppressor in lung cells and tissues, which has been indicated in other tumor types [15, 21, 22, 24].

### StarD13 is necessary for lung adenocarcinoma cell motility

**Fig. 2:**
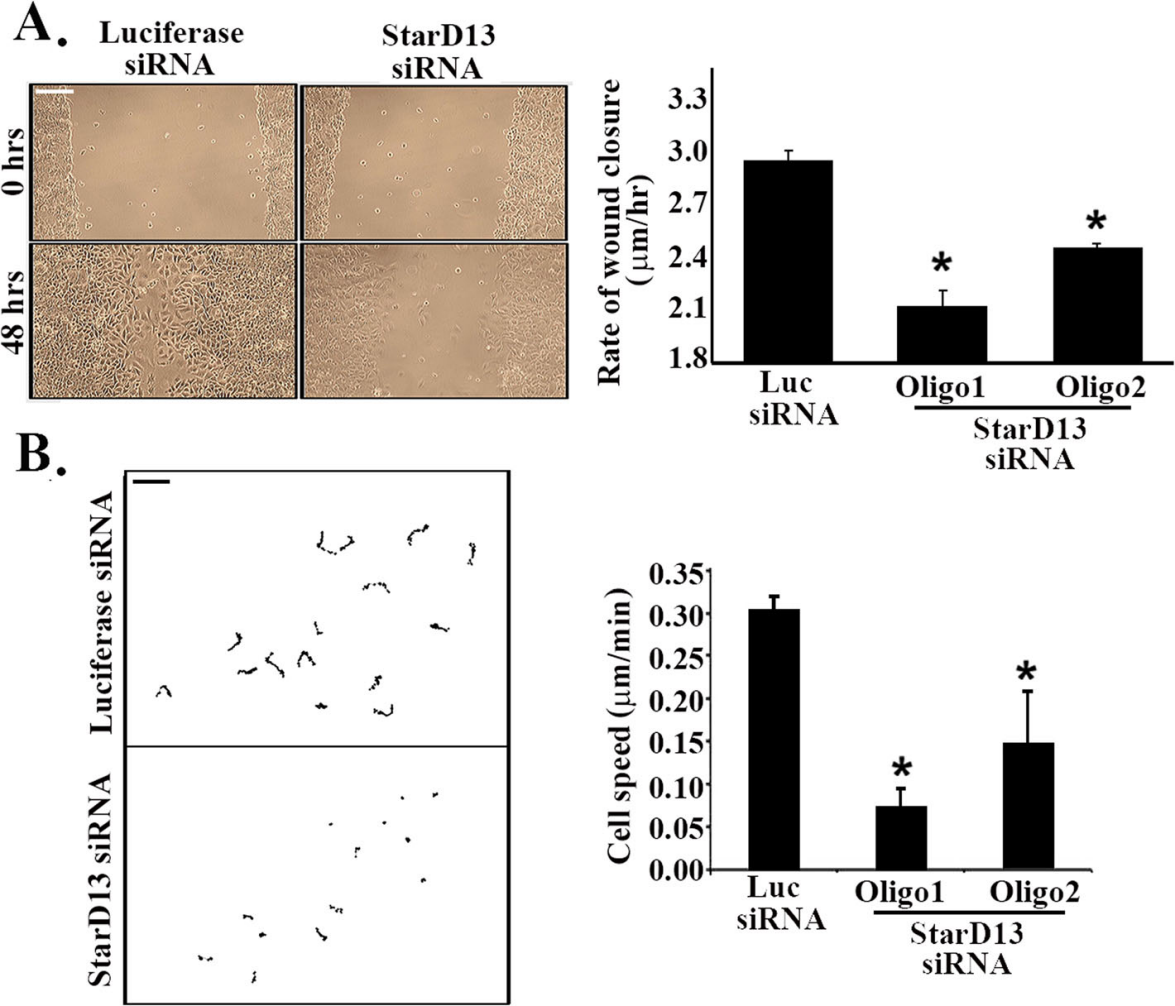
StarD13 is required for cell motility in lung adenocarcinoma. **a** A549 cells were transfected with luciferase control siRNA or with StarD13 siRNA (2 oligos) for 72 h. Cells were grown in a monolayer then wounded and left to recover the wound then imaged at the same frame after 48 h (lower micrographs). The graph is a quantitation where wound widths were measured at 11 different points for each wound, and the average rate of wound closure for the luciferase and the StarD13siRNA-transfected cells was calculated in μm/hr. Data are the mean −/+ SEM from 3 independent experiments and *p < 0.05. Scale bar is 100 μm. **b** The net paths of projected 120 frames from 2 h long time lapse movies of cells (SF268) transfected with luciferase control siRNA or with StarD13 siRNA undergoing random motility in serum (each trace is a cell). The right hand panels for each condition are a close-up trace of a cell area and a net path projected over 2 h. Graph is a quantitation of the cell speed for the time lapse movies in μm/min. Data are the mean −/+ SEM from 15 cells (from 3 independent experiments/10 movies/condition/experiment) and *p < 0.05. Scale bar is 20 μm

After determining the anticancer potential of StarD13 in A549 lung cancer cells, we assessed the molecule’s ability to modulate cell motility. To this aim, we compared the motility of control and StarD13 depleted cells in 2D using wound healing and time lapse assays. Specifically, we calculated the rate of wound closure and the individual cell speed for each condition in wound healing and time lapse assays, respectively. Depleting StarD13 with either oligonucleotides significantly decreased the wound closure rate (20 to 30%) as compared to the control (Fig. 2a).

In line with this finding, time lapse analysis revealed that both the net paths traveled by lung cancer cells (Fig. 2b) and the average speed of individual cells were reduced upon knocking down StarD13. Specifically, cells average speed decreased from 0.3 μm/min for the control, to 0.06 μm/min for cells transfected with oligo 1, and 0.15 μm/min for cells transfected with oligo 2, respectively (Fig. 2b). Oligo 1 higher efficiency of knock down thus translated in more pronounced effect on cell motility (Fig. 2a and b). Supplemental movies S1 and S2 are representatives of luciferase siRNA for oligo 1 StarD13 siRNA-transfected cells undergoing random migration in serum.

### StarD13 regulates cell motility by modulating RhoA and Rac1 activation

**Fig. 3:**
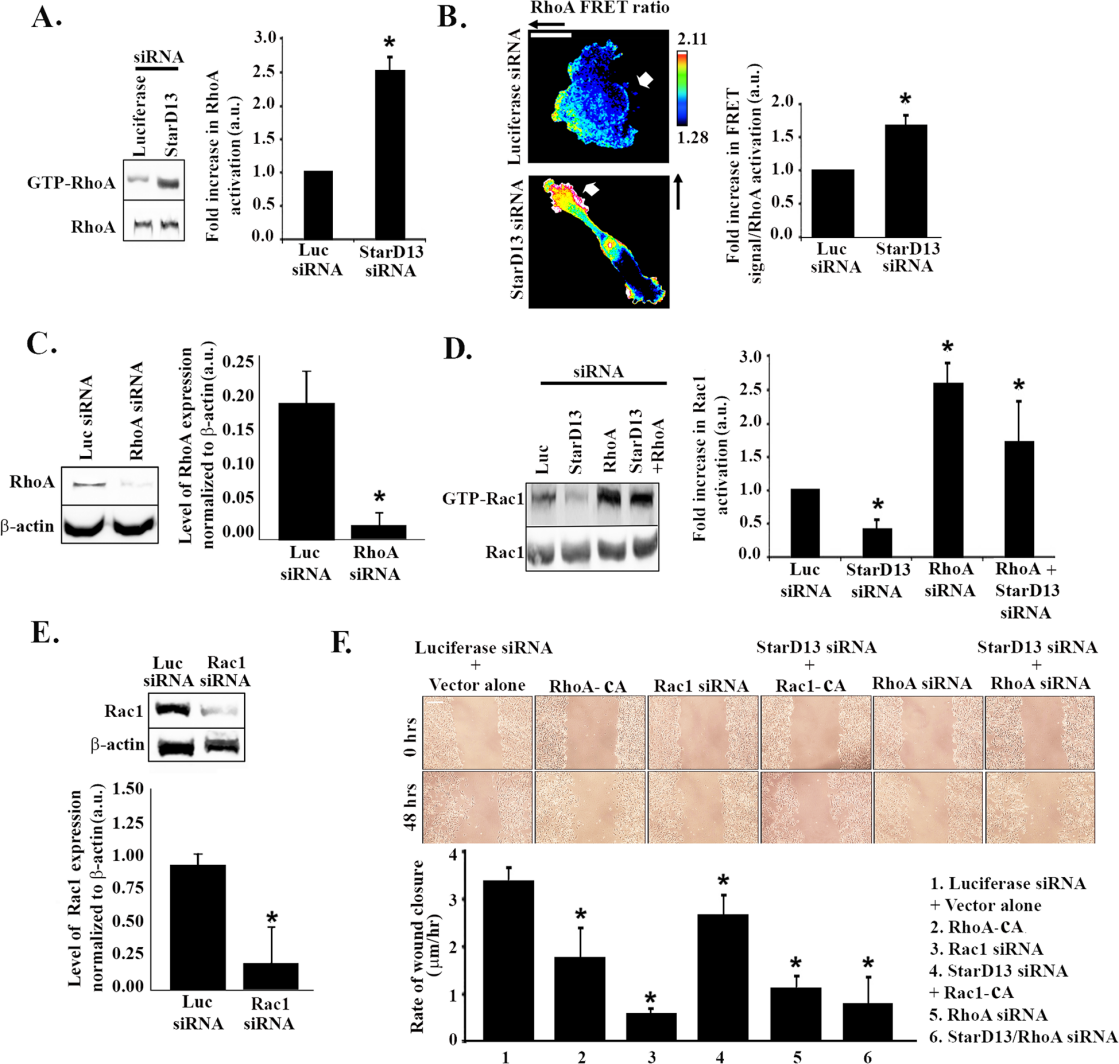
StarD13 silencing inhibits lung adenocarcinoma motility through the constitutive activation of RhoA and inhibition of Rac1. **a** A549 cells were transfected with either luciferase as control or StarD13 siRNA. After 72 h, cells were lysed and incubated with GST-RBD (Rhotekin binding domain) to pull down active RhoA. Samples were then blotted with RhoA antibody. The lower gel is a western blot for the total cell lysate, used as a loading control. The graph is a quantitation of the active RhoA bands using the ImageJ software. The bands were normalized to the amount of total proteins and the data presented as fold change to luciferase control. Data are the mean from 3 experiments −/+ SEM and *p < 0.05. **b** Representative micrographs of A549 cells transfected with luciferase or StarD13 siRNA and with the RhoA FRET biosensor and fixed. The cells were then imaged in CFP, YFP and FRET channels and image analysis performed as described in materials and methods to obtain the FRET ratios (RhoA activation). The graph is a quantitation of the FRET signal (Ratio FRET/CFP signal) in the total cell area presented as fold difference to luciferase control. Data are the mean −/+ SEM from 15 cells (from 3 independent experiments) and *p < 0.05. Scale bar is 10 μm. **c** A549 cells were transfected with luciferase control siRNA or with RhoA siRNA for 72 h. The **c**ells were then lysed and immunoblotted for RhoA or β-actin (lower gel) for loading control. The graph is a quantitation of the bands from the RhoA gel, using the imageJ software, normalized to the corresponding bands in the actin gel. Data are the mean −/+ SEM from 3 different experiments and *p < 0.05. **d** A549 cells were transfected with either luciferase, StarD13, RhoA, or StarD13 + RhoA siRNA. After 72 h, cells were lysed and incubated with GST-CRIB (Cdc42 and Rac interactive binding protein) to pull down active Rac1. Samples were then blotted with Rac1 antibody. The lower gel is a western blot for the total cell lysate, used as a loading control. The graph is a quantitation of the active Rac1 bands using the ImageJ software. The bands were normalized to the amount of total proteins and the data presented as fold change to luciferase control. Data are the mean from 3 experiments −/+ SEM and *p < 0.05. **e** A549 cells were transfected with luciferase or Rac1 siRNA for 72 h. The **c**ells were then lysed and immunoblotted for Rac1 or β-actin (lower gel) for loading control. The graph is a quantitation of the bands from the Rac1 gel, using the imageJ software, normalized to the corresponding bands in the actin gel. Data are the mean −/+ SEM from 3 different experiments and *p < 0.05. **f** A549 cells were transfected with luciferase siRNA + pcDNA3.1 vector, RhoA-CA (constitutively active), Rac1 siRNA, StarD13 siRNA + Rac1-CA (constitutively active), RhoA siRNA, and StarD13 + RhoA siRNA. Cells were then grown in a monolayer, wounded and left to recover the wound then imaged at the same frame after 48 h (lower micrographs). The graph is a quantitation of the wounds in F. Wound widths were measured at 11 different points for each condition and the average rate of wound closure was calculated in μm/hr. Data are the mean −/+ SEM from 3 wound closure assays and *p < 0.05. Scale bar is 100 μm

Previous groups have established that StarD13 is a GAP for RhoA and Cdc42 but not for Rac1 [15, 18, 21–24]. Mechanistically, StarD13 overexpression inhibited stress fiber formation [21], by modulating the downstream effectors of RhoA: ROCK and mDia. Consistently, we confirmed that StarD13 is GAP for RhoA by pull down and FRET assays. The pull down results presented in Fig. 3a and d, show that transfecting A549 lung cancer cells with StarD13 siRNA increases total RhoA activation (2.5 fold) while decreasing Rac1 activation in the cell.

Similarly, the collective FRET ratio (FRET/CFP) measured in single cells transfected with the RhoA effector-based unimolecular FRET biosensor [23, 26] show a significant increase in RhoA activation (1.6 fold) upon StarD13 knock down (Fig. 3b).

Based on the antagonistic relationship between RhoA and Rac1activation in response to StarD13 depletion, we hypothesized that the effect of StarD13 on Rac1 could be the indirect consequence of RhoA activation. Cells depleted of both StarD13 and RhoA confirmed this hypothesis. Indeed, the loss of RhoA prevented the decrease in Rac1 activation in response to StarD13 depletion (Fig. 3d). The efficiency of depleting RhoA in these cells, using siRNA, is illustrated in Fig. 3c.

This data suggests that the effect of StarD13 depletion on cell motility is mediated through the direct increase in RhoA activation, the subsequent decrease in Rac1 activation, or both. Therefore, A549 cells were transfected with a constitutively active RhoA construct or with Rac1 siRNA (proof of Rac1 depletion in these cells is illustrated in Fig. 3e) (Fig. 3f). Wound healing assays showed a significant inhibition in the migration of cells transfected with either the constitutively active RhoA construct or with Rac1 siRNA. To confirm that depletion of StarD13 is affecting motility through overactivation of RhoA and/or inhibition of Rac1, we expressed either a constitutively active Rac1 construct or RhoA siRNA oligos in StarD13 siRNA-transfected cells. Figure 3f shows that the decrease in cell motility caused by the depletion of StarD13 was partially reversed by the expression of the constitutively active Rac1 (Fig. 3f). This indeed confirms that the constitutive activation of RhoA triggered by the absence of StarD13 is inhibiting cell motility through the inhibition of Rac1. In contrast, transfecting the cells with siRNA against both RhoA and StarD13 did not restore normal cell migration. Finally, transfecting the cells with RhoA siRNA alone also inhibited cell migration, similarly to that of RhoA overactivation (Fig. 3f).

Altogether, our findings are in line with previous reports in astrocytoma and breast cancer models that uncovered the need for RhoA activation to cycle in intensity and thus allow cell migration to occur [22, 23].

### Roles of RhoA and Rac1 in lung adenocarcinoma cell adhesion dynamics

**Fig. 4:**
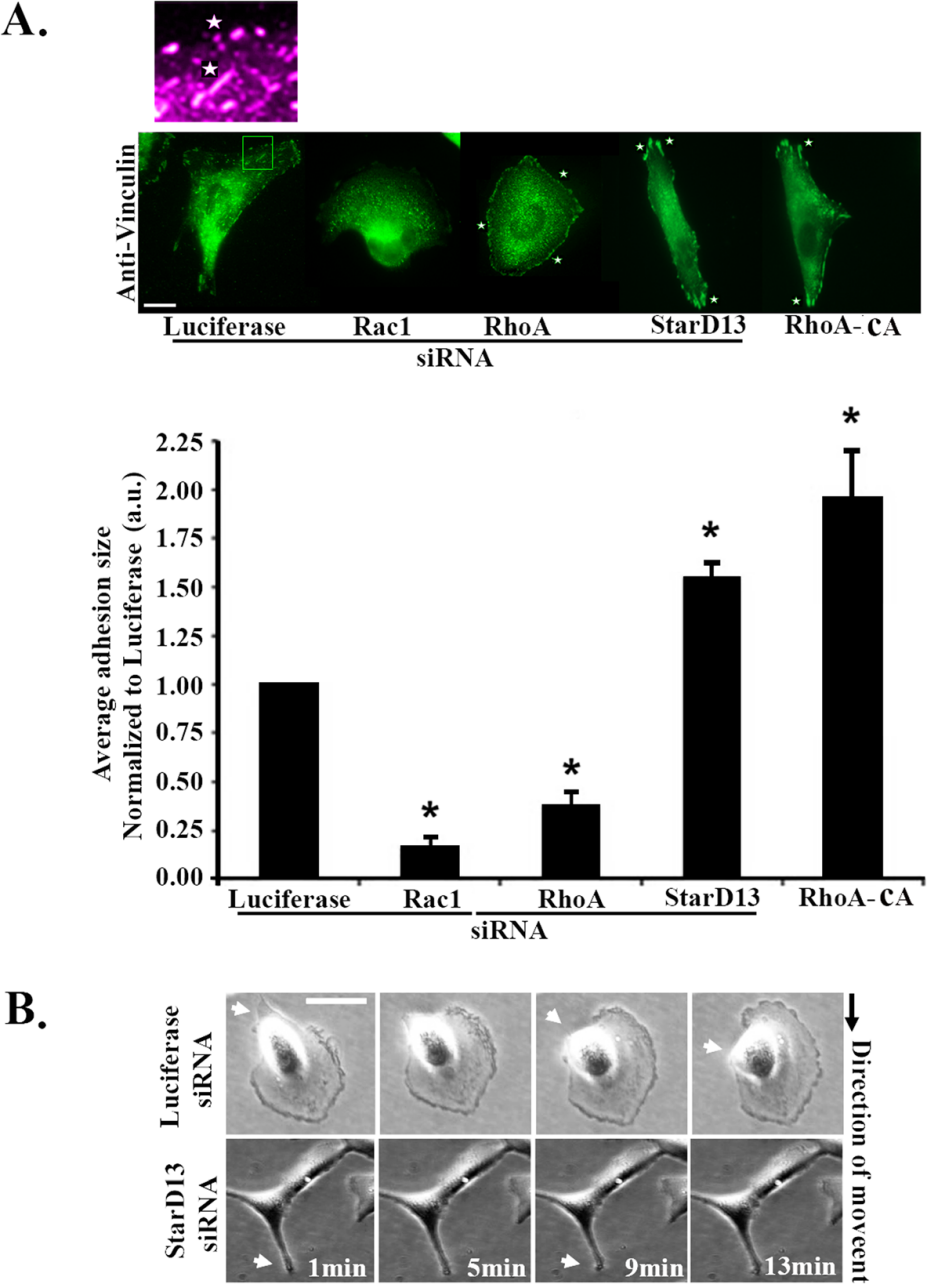
StarD13 silencing increases the size of adhesion structures in lung adenocarcinoma cells and inhibits their tail detachment. **a** Representative micrographs of A549 cells transfected with luciferase control, Rac1 siRNA, RhoA siRNA, StarD13 siRNA or RhoA-CA (constitutively active) that were fixed and immunostained with anti-Vinculin. Scale bar is 10 μm. The graph is a quantitation (as described in the materials and methods) represented as average adhesion size in cells of each condition presented as fold difference to luciferase control. Data are the mean −/+ SEM from 15 cells/condition from 3 different experiment and *p < 0.05. **b** Montage of time-lapse movie (showing frames that are 4 min apart) of Luciferase siRNA- or StarD13 siRNA-transfected A549 cells undergoing random motility in serum (for 2 h). The black arrows indicate the direction of movement. Scale bar is 10 μm

Rac1 and RhoA regulation of focal complexes assembly and maturation into focal adhesions is required for the effective cell migration of different tumor models [10, 22, 23, 31]. Therefore we sought to elucidate the mechanism through which the StarD13/RhoA/Rac1 pathway regulates cell migration in lung adenocarcinoma cells, by examining its effects on cell adhesion dynamics. Transfection of A549 cells with Rac1siRNA abolished all forms of adhesions (Fig. 4a). In addition, cells with RhoA depletion, lacked mature focal adhesions. Only punctate small adhesions, which likely represent immature focal complexes, were detected in these cells (Fig. 4a). The increase in focal complexes in the RhoA-depleted cells could be a consequence of their lack of maturation into focal adhesions or of the increase in Rac1 activation, which is a by-product of RhoA depletion as seen in Fig. 3d. This phenotype was correlated with the loss of motility in RhoA depleted cells, which suggests that focal complexes alone are not enough for the cells to move and that RhoA activation is needed for cell motility (Fig. 4A and Fig. 3F).

When cells were transfected with a constitutively active RhoA construct, the size of focal adhesions significantly increased (Fig. 4a). This was also consistently seen in cells transfected with StarD13 siRNA (due to persistent RhoA activation). We have previously shown that the inactivation of RhoA is essential for the disassembly of focal adhesions at the cell tail as well as the completion of the motility cycle [22, 23]. This was also evident in these cells as seen in Fig. 4b (and Supplemental movies S1 and S2). Indeed, while control cells detach their tail to move forward, StarD13 siRNA-transfected cells showed an elongated phenotype and were unable to detach their tail (Fig. 4b lower panel). This correlated with a high and persistent RhoA activation at the tail (defined by the direction of migration) as seen in the StarD13-depleted cells transfected with the RhoA FRET biosensor (Fig. 3b) as compared to control cells, which completely lacked RhoA activation at the cell tail during detachment.

### StarD13 silencing promotes Cdc42-mediated lung adenocarcinoma cell invasion

**Fig. 5:**
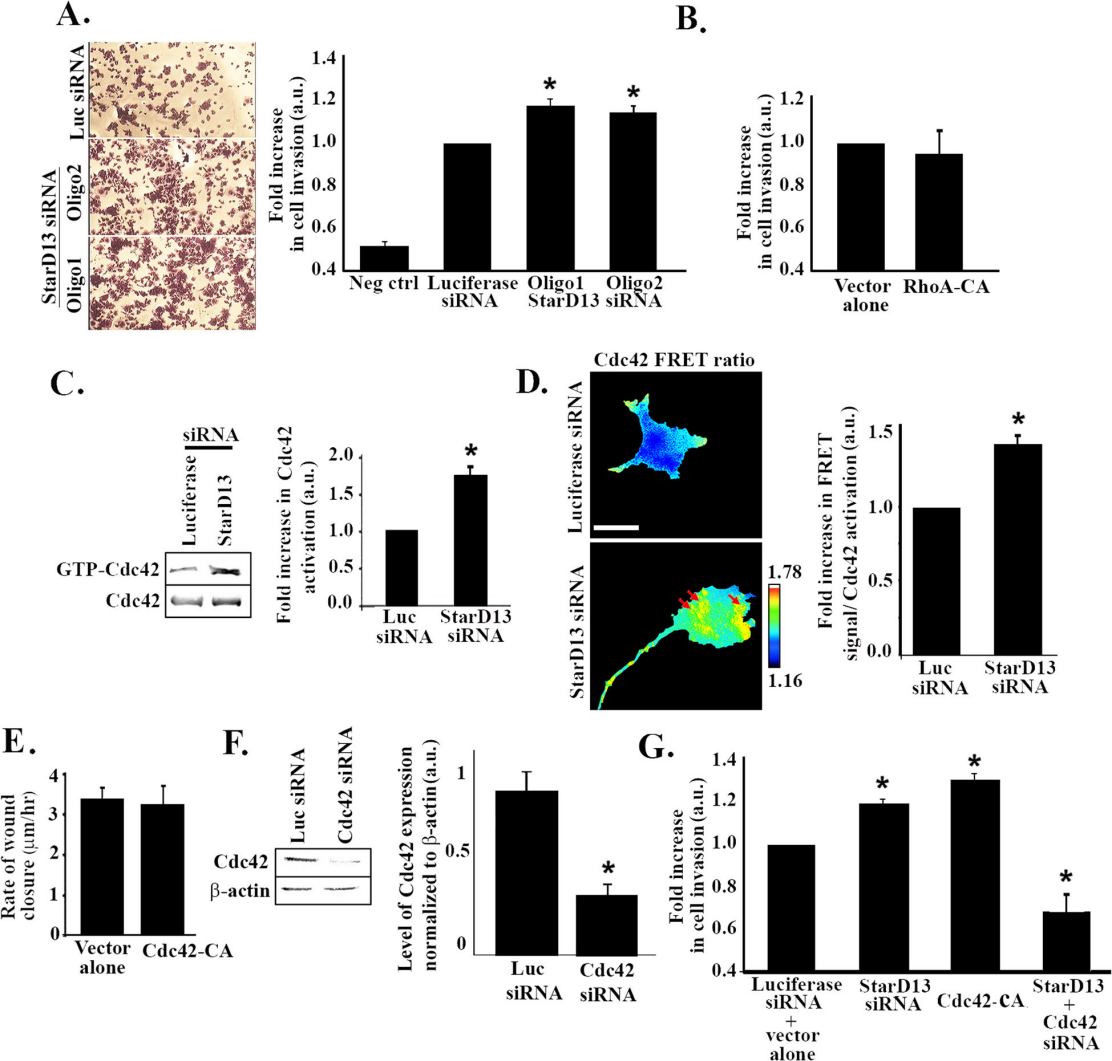
StarD13 silencing increases lung adenocarcinoma cell invasion through an increase in Cdc42 activation. **a** A549 cells were transfected with luciferase siRNA or StarD13 siRNA (2 oligos) for 72 h. Cells were then allowed to invade towards 10% FBS for 24 h. 1 × 106 cells/ml were used in each assay. The micrographs are representatives of invaded cells on the bottom side of the membrane stained with cell stain according to assay instructions. Cell stain was then extracted and colorimetric measurements were taken at 560 μm. The graph shows the measurements. Data are the mean −/+ SEM from 3 experiments and *p < 0.05. **b** A549 cells were transfected with vector alone or with RhoA-CA. The micrographs are representatives of invaded cells on the bottom side of the membrane stained with cell stain according to assay instructions. Cell stain was then extracted and colorimetric measurements were taken. Data are the mean −/+ SEM from 3 experiments and *p < 0.05. **c** A549 cells were transfected with either luciferase or StarD13 siRNA. After 72 h, cells were lysed and incubated with GST-CRIB (Cdc42 and Rac interactive binding protein) to pull down active Cdc42. Samples were then blotted with Cdc42 antibody. The lower gel is a western blot for the total cell lysate, used as a loading control. The graph is a quantitation of the active Cdc42 bands using the ImageJ software. The bands were normalized to the amount of total proteins and the data presented as fold change to luciferase control. Data are the mean from 3 experiments −/+ SEM and *p < 0.05. **d** A549 cells were transfected with luciferase or StarD13 siRNA and with the Cdc42 FRET biosensor. The cells were then imaged in CFP, YFP and FRET channels and image analysis performed as described in materials and methods to obtain the FRET ratios (Cdc42 activation). The graph is a quantitation of the FRET signal (Ratio FRET/CFP signal) in the total cell area presented as fold difference to luciferase control. Data are the mean −/+ SEM from 15 cells (from 3 independent experiments) and *p < 0.05. Scale bar is 10 μm. **e** A549 cells were transfected with either vector alone control or with Cdc42-CA (constitutively active). Cells were then grown in a monolayer, wounded and left to recover the wound then imaged at the same frame after 48 h. The graph is a quantitation of the wounds obtained. Wound widths were measured at 11 different points for each condition and the average rate of wound closure was calculated in μm/hr. Data are the mean −/+ SEM from 3 wound closure assays and *p < 0.05**. f** A549 cells were transfected with luciferase control siRNA or Cdc42 siRNA for 72 h. The **c**ells were then lysed and immunoblotted by western blot analysis for Cdc42 (upper gel) or for β-actin (lower gel) for loading control. The graph is a quantitation of the bands from the Cdc42 gel, using the imageJ software, normalized to the corresponding bands in the actin gel. Data are the mean −/+ SEM from 3 different experiments and *p < 0.05. **g** A549 cells were transfected with luciferase siRNA + vector alone, StarD13 siRNA Cdc42-DA, or StarD13 siRNA +Cdc42 siRNA for 72 h. Cells were then allowed to invade towards 10% FBS for 24 h. Cells are then stained, stains extracted and colorimetric measurements taken at 560 μm. The graph shows the measurements. Data are the mean −/+ SEM from 3 experiments and *p < 0.05

Having uncovered the role of StarD13 in cell motility, we investigated its involvement in cellular invasion using an in vitro collagen-based invasion assay and FBS as a chemoattractant. The data showed that unlike 2D migration, StarD13 inhibits lung cancer cell invasion. Indeed, StarD13 siRNA (both oligos) significantly increase cell invasion (Fig. 5a) consistent with StarD13 role as a tumor suppressor as well as previously published findings in breast cancer cell model [22]. Transfecting the cells with a constitutively active RhoA mutant however did not mimic the effect of StarD13 depletion on cell invasion (Fig. 5b) thus suggesting that StarD13 regulation of invasion in 3D is independent of RhoA activation.

Since StarD13 is a GAP for the RhoGTPase Cdc42, we investigated the contribution of Cdc42 to StarD13 regulation of cell invasion. Figure 5c shows thatStarD13 depletion significantly increases total Cdc42 activation in A549 cells, thus confirming its role as a GAP for Cdc42 in these cells. The pull down assay findings were also corroborated by FRET analysis. Indeed, the collective FRET ratio (FRET/CFP) measured upon transfection of A549 cells with a recently developed Cdc42 FRET biosensor [27], reveal a significant increase in Cdc42 activation upon StarD13 silencing (1.5 fold) (Fig. 5d).

While having no effect on cell migration (Fig. 5e), Cdc42 directly regulated invasion in A549 cells. Transfecting the cells with a constitutively active Cdc42 construct mimicked the increase in cell invasion seen in StarD13-depleted cells (Fig. 5g). This suggests that StarD13 regulation of cell invasion involves Cdc42 inhibition. Transfecting StarD13-depleted cells with Cdc42 siRNA reversed the increase in cell invasion and, significantly decreased the level of cell invasion compared to the control (Fig. 5g and Fig. 5f for Cdc42 proof of siRNA efficiency).

### StarD13 silencing promotes Cdc42-mediated invadopodia formation in lung adenocarcinoma

**Fig. 6:**
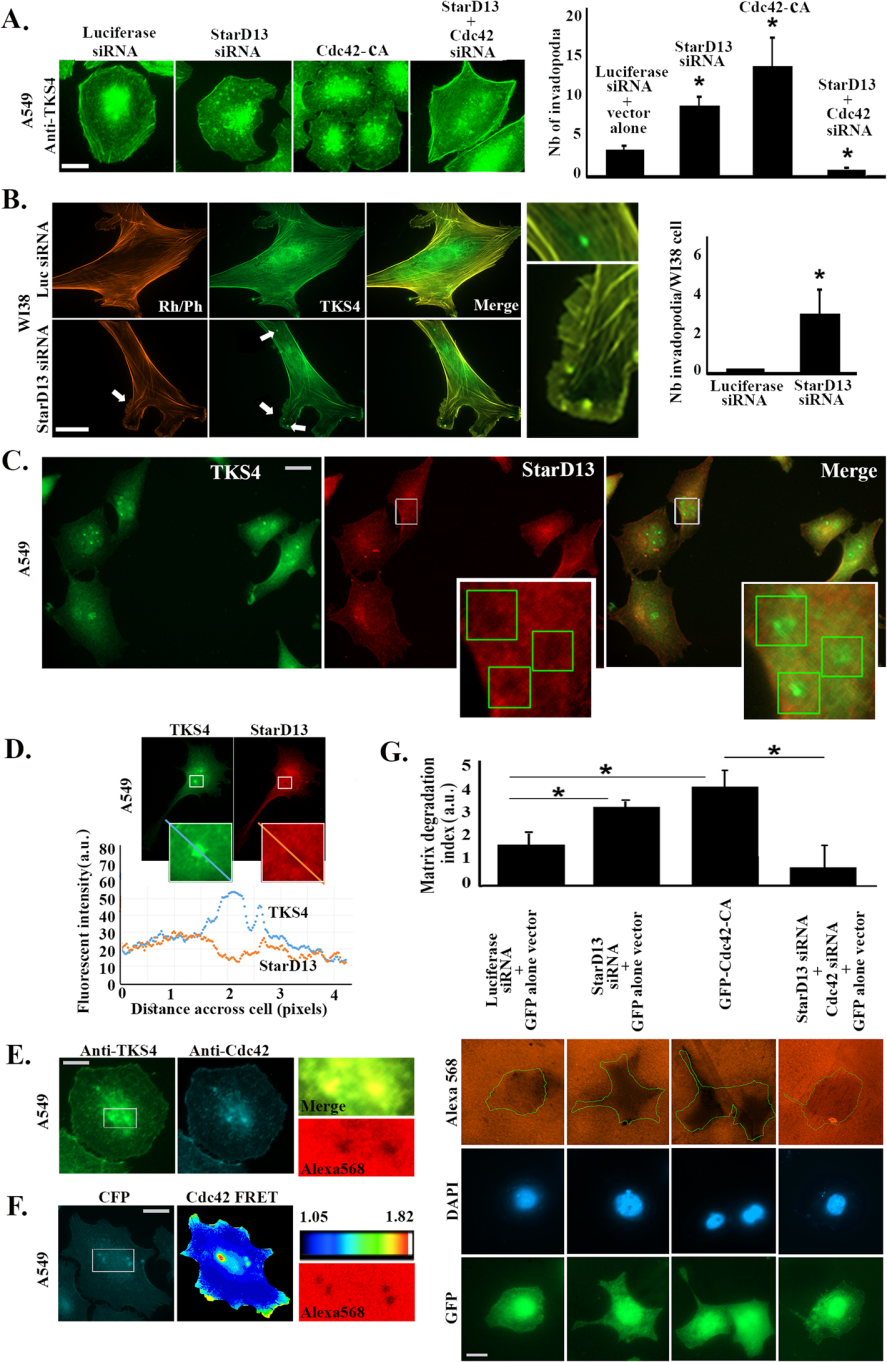
StarD13 silencing increases Cdc42-mediated invadopodia formation in lung adenocarcinoma cells. **a** Representative micrographs of A549 cells cells **t**ransfected with luciferase siRNA + vector alone, StarD13 siRNA, Cdc42-CA (constitutively active Cdc42), or StarD13 siRNA +Cdc42 siRNA for 72 h, fixed and immunostained for TKS4. Scale bar is 10 μm. The graph is a quantitation of the number of invadopodia per cell (described in materials and methods) expressed as values in every condition (15 cells/condition/experiment). Data are the mean of +/− SEM of 3 independent experiments and *p < 0.05. **b** Representative micrographs of WI38 cells transfected with luciferase or starD13 siRNA, fixed and immunostained for TKS4 and stained with Rhodamin-Phalloidin. Scale bar is 10 μm. The graph is a quantitation of the number of invadopodia per cell expressed as values in every condition. Data are the mean of +/− SEM of 3 independent experiments and *p < 0.05. **c** Representative micrographs of control A549 cells fixed and immunostained for TKS4 and StarD13. Scale bar is 10 μm. **d** Representative histogram of fluorescence intensity across the indicated lines within the cell (across invadopodia) stained for TKS4 and StarD13. The intensity is plotted as a function of distance (in pixels). **e** Representative micrographs of control A549 cells plated on Alexa568-labeled matrix for 8 h. Cells were then fixed and stained for TKS4 and Cdc42 as well as the Rhodamin channel for matrix degradation. Scale bar is 10 μm. **f** Representative micrographs of control A549 cells untreated and transfected with the Cdc42 FRET biosensor for 24 h then plated on Alexa568-labeled matrix for 8 h. Cells were then fixed and imaged in CFP, YFP and FRET channels to obtain the Cdc42 FRET signal as well as the Rhodamin channel for matrix degradation. Scale bar is 10 μm. **g** Representative micrographs of cells transfected with luciferase or StarD13 or StarD13+ Cdc42 siRNA along with a GFP vector, or with GFP-Cdc42-Q61L, or with StarD13 siRNA + GFP-Cdc42-Q61L. siRNA + GFP vector alone, StarD13 siRNA Cdc42-DA, or StarD13 siRNA +Cdc42 siRNA and plated on Alexa568-labeled matrix for 48 h. Cells were then fixed and immunostained for TKS4. Scale bar is 10 μm. The graph is a quantitation matrix degradation index expressed as ratio of mean fluorescent intensity in an ROI in the background to mean fluorescent intensity in the cell trace (as described in materials and methods). Data are the mean −/+ SEM from 3 experiments and *p < 0.05

Cdc42 activates cell invasion by stimulating invadopodia formation [32]. StarD13 is a Rho GAP for Cdc42, thus we investigated the association between StarD13, Cdc42 and invadopodia formation. To this aim, A549 cells were transfected with StarD13 siRNA or with Cdc42-DA and stained for TKS4 (an invadopodia marker). The micrographs and the invadopodia quantitation in Fig. 6a demonstrate the dramatic increase in invadopodia formation in StarD13 depleted cells and cells expressing the Cdc42-DA as compared to the control Consistently, Fig. 6a showed that invadopodia formation was completely abolished in StarD13/Cdc42 double knockdown cells, which correlated with the decrease in invasion in these cells (Fig. 5g).

Consistently, TKS4 staining showed that depleting StarD13 in WI38 normal lung cells led to the appearance of invadopodia, whereas invadopodia were completely absent in the control cells (Fig. 6b). Rhodamin Phalloidin staining also revealed the difference in the phenotypes between the control normal cells and the StarD13-depleted cells. Upon depletion of StarD13, normal cells also formed actin-rich protrusion structures indicative of an acquired migratory/invasive ability of these cells (Fig. 6).

Moreover, immunostaining for StarD13 revealed its absence from invadopodia (Fig. 6c and d). This was also reflected by the histogram in Fig. 6d (lower panel) which showed an increase in fluorescent intensity in TKS4 and a decrease in StarD13 staining across the invadopodia area. In contrast, Cdc42 located inside the invadopodia of A549 cells. Specifically, Cdc42 colocalized with TKS4 and inside the dark spots in the matrix, which result from matrix degradation ((Fig. 6e). Cdc42 activation also localized to the invadopodia as seen in Fig. 6f (red color in the FRET image coincided with the sites of invadopodia formation seen by lack of fluorescence in the Alexa channel).

To further understand the relation between StarD13, invadopodia formation and matrix degradation, A549 cells were plated on a fluorescently labeled matrix and allowed to adhere either for 8 h before monitoring for individual nascent invadopodia formation ((Fig. 6e and f), or for 48 h to look at the total degradation of the matrix (Fig. 6g). Matrix degradation measurement was obtained by tracing the cells in GFP, thresholding, and then transferring the traces to the Alexa frames. The mean fluorescent intensity within the cell area measured before expressing the matrix degradation index as a ratio of the fluorescent intensity in the background (a selected region of interest) and in the cell trace. A ratio higher than one reflected the degradation ability of the cells. A549 cells transfected with StarD13 siRNA showed an increase in matrix degradation ability (Fig. 6g). This was mimicked when cells were transfected with GFP-Cdc42-Q61L (GFP to detect transfected cells) and reversed in cells transfected with StarD13 and Cdc42 siRNA (Fig. 6g).

Collectively this data confirms that StarD13 is a tumor suppressor in lung adenocarcinoma and uncovers a RhoA-dependent mechanism for regulating cell migration and adhesion. More interestingly, this study showed, for the first time, that StarD13 negatively regulates invadopodia formation and invasion by inhibiting Cdc42 activation.

## Discussion

StarD13 is a Rho GAP specific for both RhoA and Cdc42. Previous studies have shown that StarD13 inhibits RhoA in hepatocellular carcinoma cells [18]. Consistent with its role as a tumor suppressor, StarD13 also decreased cell motility in hepatocellular carcinoma [33], and localized to focal adhesions in HeLa cells [34]. We have confirmed the tumor suppressor function of StarD13 in a wide array of cancers including, breast cancer, colon cancer and astrocytoma [21–24]. Other groups have also reported low expression levels of StarD13 in in several tumor types, due to gene deletion or epigenetic modifications [15, 20–22, 24, 35–44]. Similarly, this study uncovered that StarD13 expression levels in human NSCLC adenocarcinoma tissues are low as compared to its expression levels in tissues from normal biopsies. In line with its role in other solid tumors, the increase in cell viability and cell division in vitro upon silencing of StarD13 in lung normal and cancer cells further suggesting the potential tumor suppressor role of StarD13 in lung adenocarcinoma [17].

We have previously investigated the role of StarD13 in cancer cell motility and, revealed that despite its anti-metastatic functions the StarD13 tumor suppressor is required for cell migration [22, 23]. This was also supported by this study that demonstrates that StarD13 positively contributes to the migration of lung cancer cells. Our findings are contradictory to reported by other groups where StarD13 was found to negatively regulate the motility of cancer cells [33, 45]. However, we believe that this discrepancy could be due to the distinct role that the RhoA-governed adhesion turnover might exert on migration in different cell types. Indeed, the inhibition of cell migration in glioblastoma has been linked to aberrant increase in RhoA activity, faulty stress fiber formation and adhesion dynamics which in turn immobilize the cells in a similar manner to the effect we see in this study and others upon StarD13 depletion (and RhoA overactivation) [46]. Moreover, DLC1, a closely related protein to StarD13 and a well-established tumor suppressor, which inhibits proliferation and induces apoptosis [35], has also been shown to positively regulate cell migration [47, 48].

Consistent with the literature [21–24], we confirmed the function of StarD13 as a GAP for both RhoA and Cdc42. Consequently, we sought to determine which of the RhoGTPase mediates the effect of StarD13 knock down on cell migration. To this aim, we transfected StarD13-depleted lung adenocarcinoma cells with a constitutively active RhoA or a constitutively active Cdc42 construct. The data showed that only the constitutively active RhoA construct mimics the effect of the StarD13 depletion and decreases cell motility. This mechanism of regulation of cell motility by RhoA activation has been reported before in different systems [22, 23, 49–52]. One explanation for the mechanism underlying cell motility regulation could be, in part, due to cell paralysis in response to the disruption of adhesion dynamics upon RhoA hyperactivation. Cell migration is a dynamic process which requires all its regulatory molecules to undergo cycles of activation and inactivation. This was previously reported for RhoA activation in astrocytoma [23].

In parallel, Rac1 activation increased following the overexpression of StarD13. This was consistent with the antagonistic relationship between RhoA and Rac1 observed in other tumor types [22, 23, 26]. Hence, this suggested that the inhibition of lung adenocarcinoma cell motility in StarD13-depleted cells could be through the indirect inhibition of Rac1, caused by the constitutive activation of RhoA. Indeed, silencing Rac1 inhibited cell migration. Conversely, the transfection of StarD13 depleted cells with active Rac1 restored cell motility to normal levels. Collectively, these observations demonstrate that the constitutive activation of RhoA in the absence of StarD13 inhibits cell motility either directly or indirectly through inhibiting Rac1 or a combination of both. We had also previously shown that the silencing StarD13 (following constitutive RhoA activation) inhibits cell migration by preventing the disassembly of focal adhesions and thus stabilizing them [22, 23]. Specifically, an increase in focal adhesions inhibits cell migration by conferring the cells with more strength for attachment to the extracellular matrix [53]. At the tail, RhoA inactivation and the disassembly of adhesion complexes remains the rate-limiting step for cell migration [54]. Time-lapse movies of StarD13 depleted cells showed that a large proportion of cells unable to move forward were also unable to retract their tail. FRET analysis further revealed that these observations were accompanied by a persistent activation of RhoA at the cell tail. Hence, StarD13 inhibition of RhoA in focal adhesions at the tails is critical for cell tail detachment and their cells ability to pull themselves and move forward. Immunostaining analysis revealed that StarD13 silencing results in the formation of large and thick focal adhesions, which are potentially the major event responsible for impeding cell motility. Similar to StarD13 depletion, constitutively active RhoA also triggered the formation of larger focal adhesions. This proves that StarD13 silencing mimics the effect of constitutively active RhoA on adhesion in addition to cell motility.

RhoA plays a major role in the maturation of focal complexes into focal adhesions and the stabilization of cell protrusions. Focal complexes are small punctate structures, found behind the front of the lamellipodium [31]. On their own, focal complexes do not confer enough contractility the cells to move [31]. Focal complexes however, are precursors of focal adhesions, which appear larger in size, are less persistent [31], and provide the necessary mechanical strength for the cell bodies to contract and move forward [4]. The RhoA-dependent conversion of focal complexes into focal adhesions remains secondary to Rac1 initial triggering of the formation of these focal complexes. This has been confirmed in our study where we showed the absence of both focal complexes and focal adhesions when Rac1 is silenced and is consistent with previous studies in other cell types [22, 23, 26]. On the other hand, cells showed exaggerated focal complexes with a complete absence of focal adhesions when RhoA was silenced. In fibroblasts for instance, initial cell spreading was associated with the formation of focal complexes and with high levels of Rac1 activation [31, 55]. It is well established that the formation of focal complexes stabilizes the lamellipodium of migrating cells by binding to the ECM [5]. Consistently, some studies described a high FAK activation during initial cell spreading, which potentiates Rac1 activation and suppresses RhoA activation [56, 57]. The decrease in RhoA activity at this stage, suggests a sequential role for Rac1 and RhoA in adhesion, whereby RhoA activation is suppressed to allow Rac1-dependent focal complex formation and cells spreading. This is followed by high RhoA activation, which leads to the maturation of focal complexes into focal adhesions.

After determining the potential mechanism by which StarD13 affects random 2D cell motility, it was important to study its effect on 3D cellular invasion. Surprisingly, StarD13 silencing impaired cell migration but increased cell invasion. This was previously observed in breast cancer cells [22] and might be explained by that the potential unconventional role that focal adhesions may play in cellular invasion and matrix degradation. This was reported by a number of studies which revealed that the degradation of the underlying ECM, in several cell lines, occurs specifically at focal adhesion sites via the proteolytic activity of matrix metalloproteases (MMPs) and not through physical tension exerted by FAs onto the matrix [58]. More importantly, StarD13 increased invasion was mediated by Cdc42 activation. Indeed, silencing of Cdc42 reversed the increase in invasion observed in the StarD13 depleted cells. In addition, Cdc42 depletion inhibited invasion in control lung adenocarcinoma cells. This is in line with Cdc42 established roles in the activation of N-WASP and ARP complexes, and the induction of actin nucleation and the subsequent assembly of invadopodia [59]. In addition, Cdc42 promotes the activation and production of metalloproteinases that degrade the extracellular matrix components facilitating the process of invasion [60]. Indeed, our data showed that StarD13 depletion increase Cdc42 activation which, in turn, increases in invadopodia formation and in matrix degradation. The appearance of invadopodia was also observed in normal lung cells depleted of StarD13. This further corroborates the tumor suppressive function of StarD13 in lung tissue, since once suppressed (where otherwise StarD13 shows high expression in normal lung cells-Fig. 1c), normal lung cells recapitulated the tumor phenotype of increased proliferation (Fig. 1f) and formation of invasive structure (Fig. 6b).

Moreover, our data showed that active Cdc42 localizes to invadopodia, while StarD13 was completely absent from these structures. StarD13 was however found in areas surrounding invadopodia thus ensuring Cdc42 activation inside invadopodia. This is closely related to a spatial regulation pattern observed by Bravo-Cordero et al., whereby p190RhoGEF is excluded from the invadopodia, keeping RhoC inactive in these structures [61].

The contradictory results obtained with regards to the role of StarD13 in cell migration versus invasion have been previously reported for other proteins, including RhoA [23, 62]. Other groups also identified StarD13 as an invasion suppressor in solid tumors. Yang et al. also showed that knocking down StarD13 enhances breast cancer cell invasion in a transwell membrane assay, through Rho GTPases [36]. This was reported in another study which further identified StarD13 as a miR-125b microRNA target and showed that StarD13 silencing (being complementary to StarD13’s 3’UTR) increases the metastatic ability of gastric cancer [63, 64]. Furthermore, recent findings have revealed that StarD13 and its competing endogenous RNA, ceRNAs-3’UTRs inhibit breast cancer metastasis by inhibiting epithelial to mesenchymal transition (EMT) [65–67]. A homozygote or heterozygote loss of StarD13 in mammary tumors linked to ErbB2 (tyrosine phosphate -receptor), increased lung metastasis in vivo as well [68], further demonstrating StarD13 invasion and metastasis suppressor functions.

**Fig. 7:**
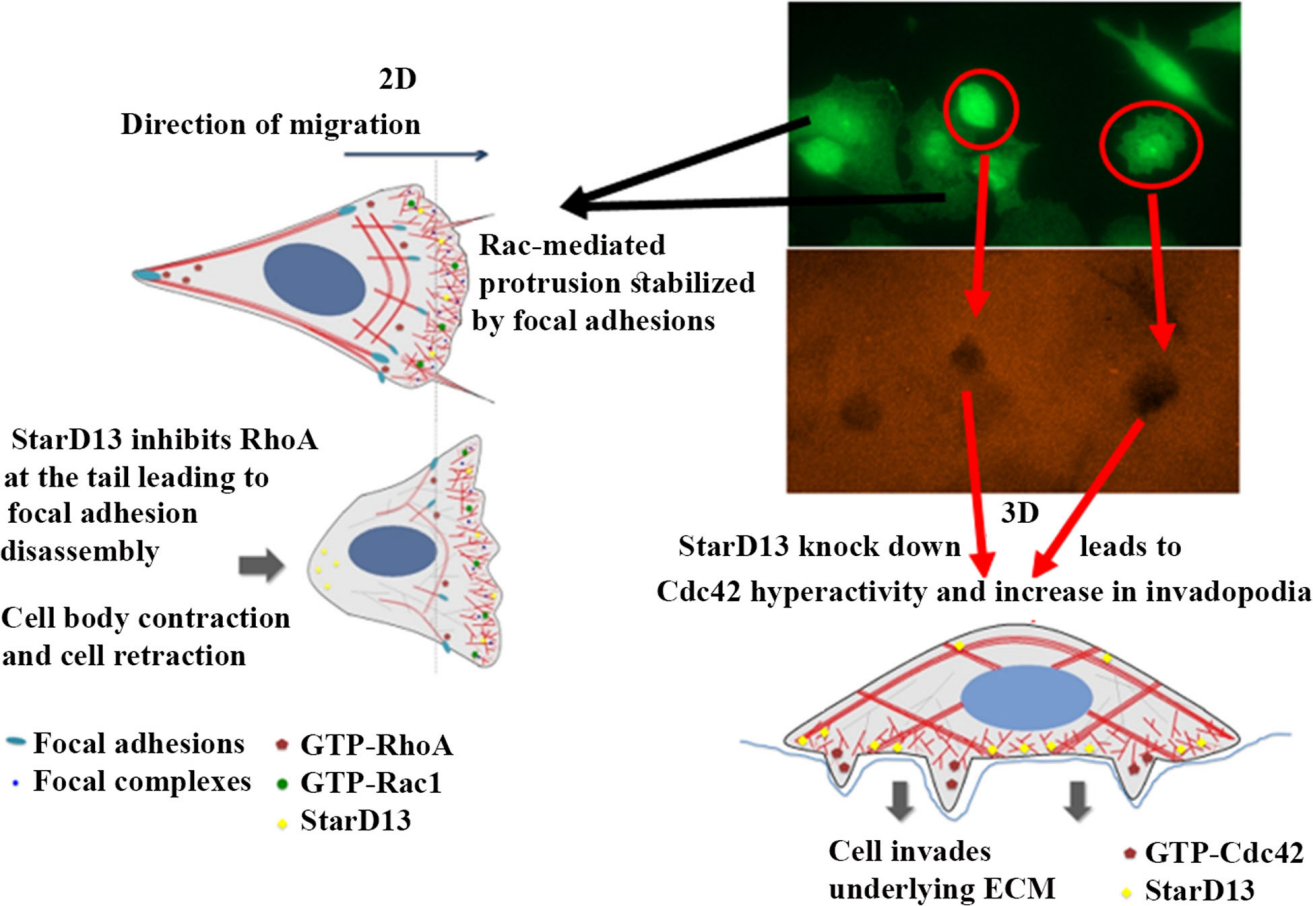
Model. The role of StarD13 in 2D and 3D migration in lung adenocarcinoma cells. The model depicts the cyclical inhibition of StarD13 of RhoA (temporal regulation) at the leading edge of migrating cells as well as the tail. This allows the cell to detach at the tail through the dissolution of focal adhesions once RhoA inactivates. At the leading edge, StarD13 inactivates RhoA which allows Rac1 to activate leading to the formation of new point contacts. RhoA then cycles back to its active form, attenuating Rac1 activity and leading to the maturating of point contacts into focal adhesions. This allows the protrusion to constructively pull the cell forward in its 2D direction. In 3D, StarD13 potentially plays a spatial inhibitory role for Cdc42 keeping its activity concentrated in invadopodia

The differential role of StarD13 in the two modes of migration (2D motility versus 3D invasion) is only indicative of cancer cells relying on different pathways for different modes of migration [69]. Thus, cells can switch between a rounded blebbing movement and a more elongated protrusive one. Thus in our study, the depletion of StarD13 increased cellular adhesion to the ECM and impeded the 2D mesenchymal cellular migration. The increase in cell movement in 3D upon StarD13 knock further suggests that cells which cannot move in an adhesion-dependent manner might switch to a more amoeboid type of movement. In conclusion, tumor cells ability to switch between modes of cell movement might limit the effectiveness of prospective inhibitory strategies targeting particular cell morphology and allow the tumors to escape inhibition. Interestingly, as shown in Fig. 7 (model), the cells that showed a mesenchymal-like phenotype (adherant, spread) had less matrix degradation ability than the cells that showed an ameboid-like phenotype (round cells), when plated on an invasive matrix.

Collectively, the data in this study confirmed that StarD13 is a GAP for RhoA and Cdc42. Specifically; the results emphasized the importance of StarD13 in attenuating RhoA activation in a temporal manner and Cdc42 activation in a spatial manner. Temporal inactivation of RhoA will allow it cycling between its active and inactive forms. This cyclical activity is essential at the edge of the cell where: 1. Rac1 activation (RhoA has to be inactive) leads to the formation of initial focal complexes. 2. RhoA activation, inactivates Rac1 (antagonistic relationship) and leads to the maturation of focal complexes into focal adhesions, thus providing enough force for the cell to fix its protrusion on the ECM and move forward. In addition, as RhoA gets inactivated, focal adhesions dissolve at the cell tail allowing the cell to move forward. Cdc42 is inhibited by StarD13 but it remains active at sites of invadopodia, where Cdc42 is required for activating nucleation promoting factors in a similar way to the spatial regulation described for RhoA and RhoC in breast cancer cells [61] (Fig. 7, model). Finally, it is well established that RhoA activates the small GTPase Ras thus stimulates cell proliferation through the mitogen-activated protein kinase kinase (MAPKK) Raf-1 (the Rapidly Accelerated Fibrosarcoma) signaling [70, 71]. This suggests a dual role of StarD13; as 1- a tumor suppressor which attenuates RhoA (hence Raf-1/MEK/ERK proliferation and survival pathway) as well as 2- an invasion suppressor, which inhibits Cdc42.

## Conclusion

Invadopodia signaling has not yet been studied in lung cancer and StarD13 is a key regulator of RhoGTPases, hence of invadopodia. This study established StarD13 as a potential tumor suppressor in lung cancer, similarly to other solid tumors. It also examined the role of StarD13 in regulating lung cancer cell migration and invasion through the regulation of RhoGTPases. Stard13 was found to be required for the cycling activation of RhoA which allows for adhesion dynamics needed in cell migration. StarD13 was also shown, for the first time, to regulate invadopodia formation through the suppression of Cdc42 activity. In summary, this study defined distinct roles of StarD13 in lung cancer cell migration and invasion through its differential regulation of Rho GTPases.

## Supplementary information

**Additional file 1: Supplemental Figure S1.** Data analyzed from Oncomine website. mRNA of the indicated number of samples (indicated in the legend for every tumor type) were quantified for expression levels of StarD13 in Garber lung (upper), Hou lung (lower left) and Su lung (lower right).

**Additional file 2: Supplemental movie S1.** A549 transfected with luciferase siRNA and undergoing random 2D migration in serum for 2 h (120 frames, a frame every minute, 40 X objective).

**Additional file 3: Supplemental movie S2.** A549 transfected with StarD13 siRNA and undergoing random 2D migration in serum for 2 h (120 frames, a frame every minute, 40 X objective).

## Data Availability

The datasets used and/or analysed during the current study are available from the corresponding author on reasonable request.
