# Supplementary figures and images for: Differential regulation of rho GTPases during lung adenocarcinoma migration and invasion reveals a novel role of the tumor suppressor StarD13 in invadopodia regulation
Source: Cell Commun Signal. 2020 Sep 8;18:144. doi: 10.1186/s12964-020-00635-5 (PMC7487901; doi:10.1186/s12964-020-00635-5)

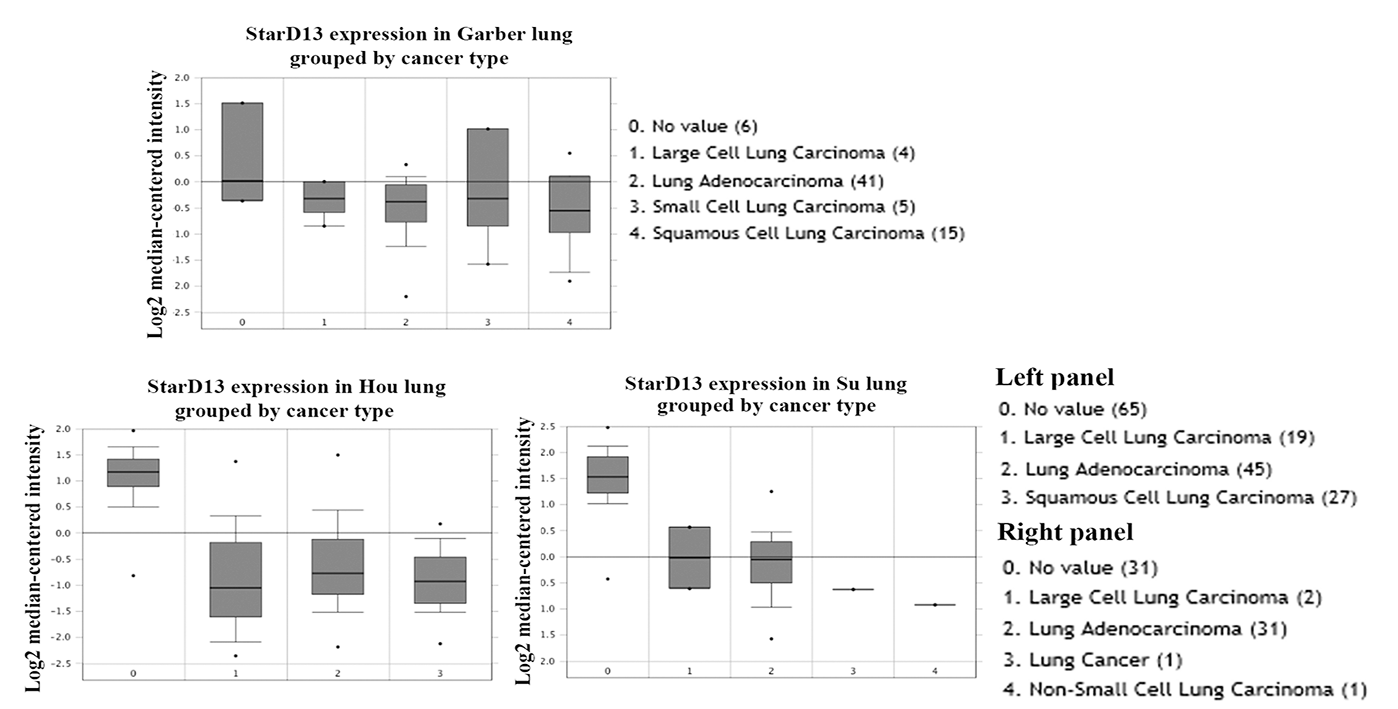

Supplement: Supplementary file 2 — Additional file 1: Supplemental Figure S1. Data analyzed from Oncomine website. mRNA of the indicated number of samples (indicated in the legend for every tumor type) were quantified for expression levels of StarD13 in Garber lung (upper), Hou lung (lower left) and Su lung (lower right). [file 12964_2020_635_MOESM1_ESM.tif]
